# Potential Effect of Statins on *Mycobacterium tuberculosis* Infection

**DOI:** 10.1155/2018/7617023

**Published:** 2018-11-15

**Authors:** Paola Del Carmen Guerra-De-Blas, Pedro Torres-González, Miriam Bobadilla-Del-Valle, Isabel Sada-Ovalle, Alfredo Ponce-De-León-Garduño, José Sifuentes-Osornio

**Affiliations:** ^1^Laboratory of Clinical Microbiology, Department of Infectious Diseases, Department of Medicine, Instituto Nacional de Ciencias Médicas y Nutrición Salvador Zubirán, Mexico City, Mexico; ^2^Laboratory of Integrative Immunology, Instituto Nacional de Enfermedades Respiratorias “Ismael Cosío Villegas”, Mexico City, Mexico

## Abstract

Tuberculosis is one of the 10 leading causes of death in the world. The current treatment is based on a combination of antimicrobials administered for six months. It is essential to find therapeutic agents with which the treatment time can be shortened and strengthen the host immune response against *Mycobacterium tuberculosis*. *M. tuberculosis* needs cholesterol to infect and survive inside the host, but the progression of the infection depends to a large extent on the capacity of the immune response to contain the infection. Statins inhibit the synthesis of cholesterol and have pleiotropic effects on the immune system, which have been associated with better results in the treatment of several infectious diseases. Recently, it has been reported that cells treated with statins are more resistant to *M. tuberculosis* infection, and they have even been proposed as adjuvants in the treatment of *M. tuberculosis* infection. The aim of this review is to summarize the immunopathogenesis of tuberculosis and its mechanisms of evasion and to compile the available scientific information on the effect of statins in the treatment of tuberculosis.

## 1. Introduction

Tuberculosis is an infectious disease caused by the *Mycobacterium tuberculosis* complex, which includes the species *M. tuberculosis*, *M. bovis*, BCG strain of *M. bovis*, *M. africanum*, *M. caprae*, *M. microti*, *M. canettii*, and *M. pinnipedii*. Of these, *M. tuberculosis* is the cause of 98% of cases in humans. The most common site of infection is the lung, although it can affect other organs and systems (lymph nodes, bones, meninges, etc.) [1]. Tuberculosis is one of the 10 leading causes of death in the world, is the leading cause of death by a single infectious agent, and produces more deaths than HIV/AIDS. In 2016, the World Health Organization reported that there were 10.3 million new cases and 1.4 million deaths caused by tuberculosis [2].

The current treatment against tuberculosis is based on the administration of a combination of antimicrobials for six months. The purpose of this strategy is to cure the disease, eradicate the infection, prevent relapse, and prevent the development of resistance. This strategy has been used for the past 60 years; however, the lengthy treatment and its adverse effects favor poor adherence, failure, and the development of resistance. Despite clinical cure, approximately half of treated patients have permanent lung damage due to excess inflammation caused by this infection [3, 4]. Therefore, it is essential to find therapeutic agents with the potential to shorten treatment time and, eventually, with the capacity to strengthen the immune response against *M. tuberculosis*.

There are two different approaches in the search for new anti-TB drugs: bactericidal antimicrobials and host-directed therapies (HDT) that improve the immune response of the host to the infection. These approaches reduce excess inflammation, prevent damage to tissues, preserve lung function, and possibly improve the effectiveness of anti-TB treatment to eliminate infection [5]. Examples of this group are vitamin D, rapamycin, and statins.

Statins inhibit the main enzyme in the synthesis of cholesterol, called 3-hydroxy-3-methylglutaryl-CoA (HMG-CoA) reductase. Its main use is in patients with hypercholesterolemia and in the prevention of cardiovascular diseases in patients with risk factors such as dyslipidemia, diabetes, hypertension, and smoking [6]. However, it has been reported that statins have pleiotropic effects on the immune system and have been associated with better outcomes in several infectious diseases. Recently, statins have been proposed as adjuvants in the treatment of *M. tuberculosis* infection [7]. Based on the above, the objective of this review is to summarize the immunopathogenesis of tuberculosis and to collect all available scientific information on the effect of statins in the treatment of tuberculosis.

### 1.1. Immunopathogenesis of Tuberculosis

Infection by *M. tuberculosis* begins with the inhalation of aerosols. Although most inhaled bacilli are trapped in the upper respiratory tract, approximately 10% of them reach the alveoli, where they are phagocytosed by cells of the innate immune response (macrophages, dendritic cells, and alveolar epithelial cells) [8]. Phagocytosis of *M. tuberculosis* involves the participation of complement receptors (CR1, CR3, and CR4), Fc, and mannose, among others.

It has been noted that the control of the infection depends to a large extent on the effective acidification of the phagosome, the adequate fusion of the phage lysosome, and the activation of processes such as autophagy and apoptosis in the abovementioned cells [9].

The recognition of *M. tuberculosis* during the innate immune response triggers cell activation and the production of cytokines and proinflammatory chemokines such as interferon- (IFN-) *γ*, tumor necrosis factor- (TNF-) *α*, interleukin- (IL-) 6, IL-12, IL-17, IL-23, C-C motif chemokine ligand (CCL)2, CCL3, CCL5, C-X-C motif chemokine ligand (CXCL)8, and CXCL10 [10, 11].

**Figure 1:**
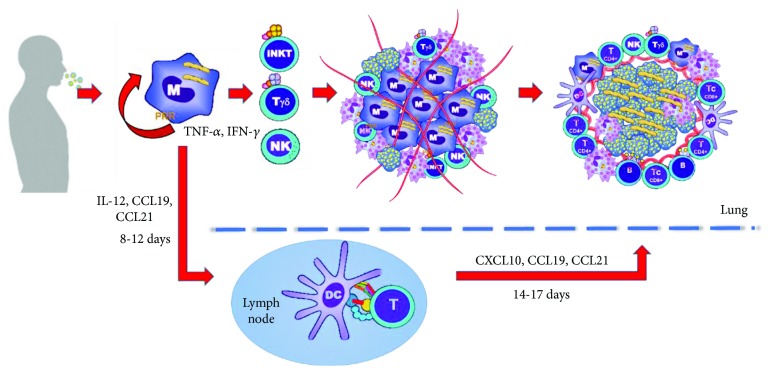
Immunopathogenesis of tuberculosis. The *M. tuberculosis* infection begins with the inhalation of airdrops that contain numerous bacilli that are phagocytosed. The initial stages of infection are directed by cells responsible for innate immunity, and the recruitment of inflammatory cells leads to the formation of an early granuloma. Antigen-presenting cells migrate to nearby lymph nodes and activate lymphocytes that return to the lung and generate the mature granulomas. The immune system contains the primary infection in almost 90% of patients, who will develop latent tuberculosis.

A key cytokine in limiting the intracellular growth of bacilli is IFN-*γ*, which is secreted in the initial stages by natural killer (NK) cells, gamma delta T cells, and natural killer T (NKT) cells (Figure 1). NK cells lyse monocytes and macrophages infected with *M. tuberculosis* through perforin-mediated cytotoxic activity, granzymes, and the Fas-FasL system [12]. Other cells that help to control the growth of *M. tuberculosis* and that contribute to the formation of the granuloma are the invariant natural killer T (iNKT) cells. The iNKT cells are important in the activation of macrophages and dendritic cells through the production of IFN-*γ*; additionally, they have been shown to be capable of killing macrophages infected with *M. tuberculosis* [13]. It has been reported that the specific activation of iNKT cells by the alpha-galactosylceramide ligand presented via CD1d protects inbred mouse strains susceptible to tuberculosis [14]. However, several studies have shown that subjects with active tuberculosis had a lower number of iNKT cells, which could indicate that they are more susceptible to *M. tuberculosis* infection [15]. IL-1*β* and IL-18 contribute to the containment of infection. They are produced by mononuclear phagocytes and promote the recruitment of neutrophils and monocytes to the site of infection [16]. Recent reports show that IL-1*β* increases the secretion and signaling of TNF, as well as the expression of TNFR1, which leads to the activation of caspase 3, apoptosis, and death of *M. tuberculosis* in macrophages [17].

**Figure 2:**
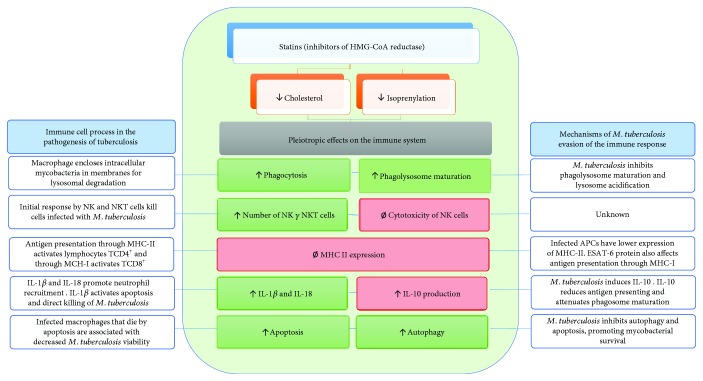
Pleiotropic effect of statins. Statins exert diverse effects on the immune response. It has been reported that statins can promote autophagy (in macrophages) and apoptosis (in tumor cells). Statins increase the number of NK and NKT cells. Statins may inhibit cytotoxicity of NK cells. Statins inhibit MHC-II expression (in CPA) and promote discrete secretion of IL-1*β*, IL-18, and IFN-*γ* (in mononuclear cells). Statins increase serum levels of IL-10. The blue boxes show the function of the immune cells in the pathogenesis and the mechanisms of *M. tuberculosis* evasion of the immune response; at the center, the blue lines demonstrate that the effect of statins is related to these processes; the green boxes show the statin effects that potentially favor the immune response against *M. tuberculosis*; and the red boxes indicate the statin effects that could modulate the immune response against *M. tuberculosis*.

The recruitment of inflammatory cells leads to the formation of early granuloma (Figure 1), composed of macrophages, dendritic cells, neutrophils, apoptotic cells, and necrotic cells [18]. Several experiments have suggested that the mechanism of apoptosis more effectively favors the elimination of bacillus, while the mechanism of necrosis favors its dissemination [19]. Fratazzi et al. reported that infected macrophages that die by apoptosis are associated with decreased mycobacterium viability, whereas this decrease is not observed if macrophages die due to necrosis (Figure 2) [20].

The development of the effector immune response by T lymphocytes requires the processing and presentation of bacterial antigens through major histocompatibility complex (MHC) molecules expressed by antigen-presenting cells (APCs). The presentation of antigens to naïve T cells occurs through MHC class II (MHCII) for CD4^+^ T lymphocytes and class I (MHCI) for TCD8^+^ lymphocytes (Figure 1) [21]. The main effector mechanism of CD4^+^ T cells is the production of IFN-*γ*, which induces the maturation and activation of macrophages to control or eliminate the bacillus; it has been observed that the depletion of CD4^+^ T cells in animal models causes reactivation of the infection and death of the host, with high bacterial loads in the lung [22]. Another function of CD4^+^ T cells is the direct production of other cytokines, such as IL-2 and TNF-*α* [23].

CD8^+^ T lymphocytes also play an important role in the cellular response against mycobacteria since they are capable of killing infected cells or of directly eliminating bacilli [24, 25].

Likewise, nonconventional T cells (*γδ* T cells) are activated and contribute to fight *M. tuberculosis* infection. For example, a marked expansion of *γδ* T cells in the blood of patients with tuberculosis has been reported. These cells contribute to the secretion of cytokines (IFN-*γ* and TNF-*α*), cytotoxic effector function, and cellular contact-dependent signaling [26].

Recently, the involvement of B cells in the process of developing immunity during tuberculosis infection has been described. These cells favor antigenic presentation and through the production of antibodies opsonize the bacilli, activate the complement, and promote the formation of memory cells and plasma cells [27].

With the formation of the granuloma (Figure 1), the immune system controls more than 90% of the bacterial load of the primary infection. In this way, most infected subjects develop latent tuberculosis (defined as a state where the individual is infected but remains free of clinical manifestations); however, if the infecting bacterial inoculum is very large, if the subject has HIV, or if the individual has some primary immunodeficiency, the infection may evolve to progressive active tuberculosis [28]. In subjects who develop latent tuberculosis, when an immunosuppressive condition appears and alters the homeostasis of the immune system, such as the coexistence of diabetes mellitus, the administration of immunosuppressive drugs, or a state of malnutrition, the bacillus is reactivated and initiates its replication, the granulomas are broken, the levels of proinflammatory cytokines increase, and the clinical manifestations of the disease appear. Therefore, active tuberculosis is defined precisely by the appearance of signs and symptoms of the disease (chronic cough, sputum, and/or hemoptysis) confirmed by the isolation of *M. tuberculosis* in the cultures [29].

### 1.2. Mechanisms of *Mycobacterium tuberculosis* Evasion of the Immune Response

*M. tuberculosis* is able to evade both innate and adaptive immune responses. In macrophages, bacteria inhibit phagosome maturation by different mechanisms, such as the retention of the protein TACO **(**tryptophan aspartate-containing protein) and the Rab5 protein in the phagosome (Figure 2) [30]. This retention prevents the process called “Rab conversion,” through which Rab5 is exchanged for Rab7, which inhibits phagolysosome fusion [31]. To perform this function, the phosphatidylinositol 3-phosphate molecule produced by hVPS34 kinase is necessary. Another mechanism of inhibition of phagolysosome maturation occurs through the glycosylated lipoarabinomannan of the *M. tuberculosis* cell wall, which decreases the activity of hVPS34 and inhibits the recruitment of the early endosomal antigen 1 (EEA1) [32].

Under normal conditions, phagocytic cells, such as neutrophils, monocytes, macrophages, and eosinophils, destroy phagocytosed microorganisms by producing reactive oxygen and nitrogen species (NO-, O2-, and ONOO-). However, *M. tuberculosis* can inhibit the recruitment of iNOS into the phagosome and prevent the formation of reactive oxygen and nitrogen species [33]. In addition, *M. tuberculosis* secretes methionine sulfoxide reductase enzymes A and B that reduce peroxynitrite (ONOO-) in nonionic molecules, which do not destroy mycobacteria [34].

Another form of immune response evasion is the inhibition of antigenic presentation. *In vitro* models have shown that macrophages infected with *M. tuberculosis* reduce the expression of MHCII [35]. Recent data from *in vivo* experiments demonstrated that APCs infected with *M. tuberculosis* have lower expression of MHCII compared with uninfected APCs [36]. It has also been reported that the ESAT-6 protein interacts with beta-2-microglobulin, which affects the function of antigen presentation through MHCI (Figure 2) [37], and it has been shown that the suboptimal presentation of the antigen contributes to the persistence of *M. tuberculosis in vivo* [38]. In addition, the inhibition of antigenic presentation has been associated with the virulence of the strains, since, for example, *M. tuberculosis* H37Rv has a greater capacity to inhibit antigenic presentation compared with H37Ra [39].

The antigenic presentation of the dendritic cells is also affected in the normal maturation process, which is essential for the proper activation of the cell [40]; in contrast, they have a lower expression of integrins, which decreases the capacity of the dendritic cells to migrate to the lymph nodes [41]. There is evidence that *M. tuberculosis* infection alters the presentation of lipid antigens by CD1 molecules. Mycobacterial wall alpha-glucan negatively regulates the expression of CD1 in APCs [42, 43]. Recent data indicate that immune evasion can occur not only through a blockade of the maturation of dendritic cells but also by facilitating poorly coordinated maturation. According to this model, *M. tuberculosis* induces the movement of MHC-II molecules to the cell membrane and inhibits the synthesis of new MHC-II-type molecules. As a consequence, the presentation of antigenic peptides of *M. tuberculosis* is affected (Figure 2) [44].

In the adaptive immune response, Mahon et al. have shown that the glycolipids of the *M. tuberculosis* cell wall, including the mannosylated lipoarabinomannan (LAM), directly inhibit the activation of polyclonal CD4^+^ T cells by blocking the phosphorylation of ZAP-70 [45], and Sande et al. reported that LAM induces CD4^+^ T cell anergy by inducing the overexpression of GRAIL (the receptor associated with the induction and maintenance of anergy in CD4^+^ T cells) [46]. Saavedra et al. showed that the activation of CD8^+^ T lymphocytes is also inhibited by the mycobacterial glycolipid 2,3-di-O-acyl-trehalose (DAT) since it reduces the cellular proliferation induced by the antigen [47].

Another way that *M. tuberculosis* evades the immune response is by altering the production of cytokines. The production of proinflammatory cytokines, such as TNF-*α*, IL-6, and interleukin-1 beta (IL-1*β*), and the chemokine MCP-1 is inhibited by phenolic glycolipids present in the cell wall of *M. tuberculosis* [48]. *M. tuberculosis* inhibits the production of IL-12, and the *mm*A4 gene has been identified as a key locus for such inhibition [49]. It has also been reported that signaling induced through TLR2 by *M. tuberculosis* in macrophages induces the secretion of IL-10, suppresses IL-12, and attenuates the Th1 response, which is critical for controlling infection [50]. Wang et al. reported that *M. tuberculosis* induces the expression of the IL-10 gene and that IL-10 reduces antigen presentation and attenuates phagosome maturation, which prevents bacterial death and induces infection by *M. tuberculosis* over the long term in the lung (Figure 2) [51].

The survival of *M. tuberculosis* within the host macrophages implies apoptosis resistance dependent on overexpression of the antiapoptotic protein Mcl-1 (Figure 2) [52]. It has also been described that *M. tuberculosis* causes a significant alteration of the inner membrane of the mitochondria of macrophages, which favors cell death by necrosis, a mechanism that promotes the spread of the pathogen and the appearance of the disease [53]. *M. tuberculosis* can evade the autophagy mechanism through the ectopic expression of ESAT-6, which inhibits the formation of autophagosomes in infected macrophages [53] and reduces the expression of Atg8 (ubiquitin-like protein) in human dendritic cells (Figure 2) [54].

### 1.3. The Role of Cholesterol in *Mycobacterium tuberculosis* Infection

The cell wall of *M. tuberculosis* contains an abundant amount of lipids, and a relatively large fraction of its genes encode proteins for their synthesis. It has been shown that mycobacteria can accumulate and use cholesterol as a carbon source and for the synthesis of some virulence factors, such as phthiocerol dimycocerosate and sulpholipid-1 [55]. It has been reported that *M. tuberculosis* dysregulates the metabolism of lipids in the host and the progression of the granuloma until the caseation correlates with the high expression of the genes for lipid metabolism and sequestration [56]; in an apoE -/- mouse model, Martens et al. studied the effect of hypercholesterolemia in tuberculosis infection, observing that hypercholesterolemic mice infected with *M. tuberculosis* have a greater bacillary load and an accentuated pulmonary pathology [57]. In another human study, it was reported that dietary cholesterol is dose-dependently associated with an increased risk of having active tuberculosis [58].

Cholesterol is essential for the internalization of mycobacteria in host cells [30]. *M. tuberculosis* enters cells through high-cholesterol domains, and by eliminating cholesterol from the cell membrane, phagocytosis is inhibited [59, 60]. Within the macrophages, the mycobacterium can inhibit the maturation of the phagosome, along with its hydrolytic and microbicidal capacities, and cholesterol also plays an important role in arresting the phagosome's maturation. For example, the accumulation of cholesterol causes abnormal retention of the TACO protein [30] and inhibits the dissociation of Rab7 from the phagosome membrane [61].

The foamy macrophages of the granulomas of patients with tuberculosis are reservoirs rich in nutrients, have altered their phagocytic capacity, and favor the persistence of mycobacteria [62]. One study shows that *M. tuberculosis* infection induces the accumulation of oxidized low-density lipoprotein in alveolar macrophages, and when alveolar macrophages are exposed to oxidized low-density lipoproteins *in vitro*, the survival and persistence of intracellular bacilli are promoted; the mechanism is unknown but could be related to the use of host cholesterol as a source of energy [63].

## 2. Pleiotropic Effect of Statins

The main mechanism of the action of statins is the inhibition of the enzyme HMG-CoA reductase, which regulates the synthesis of cholesterol and has been used mainly in patients with hypercholesterolemia. Recently, pleiotropic effects of statins on the immune system and some bactericidal effects have been reported [64] (Figure 2).

It has been documented that statins have the capacity to act as immunomodulators. For example, statins induce the phagocytic activity of macrophage J774 [65]. It has also been reported that they act as inhibitors of the expression of MHCII induced by IFN-*γ* in primary endothelial cells, monocytes, and human macrophages, which in turn inhibits the activation of T lymphocytes [66]. The treatment of mononuclear cells with fluvastatin produced the discrete activation of caspase 1 and moderated the secretion of IL-1*β*, IL-18, and IFN-*γ* [67]. It has also been demonstrated that statins upregulate IL-10 in the serum of patients with acute coronary syndrome [68].

Another study showed that the *in vitro* treatment of mononuclear cells with atorvastatin increases the number of NK and NKT cells in peripheral venous blood [69]. It has also been reported that treatment with simvastatin and IL-2 promotes the activation of NK cells [70]. In contrast, other studies have reported that statins inhibit the cytotoxicity of NK cells [71] and the function of activating receptors [72]. It has also been shown that simvastatin therapy in patients with hypercholesterolemia for six months increases the iNKT cells in peripheral venous blood [73].

Other studies show that statins can induce apoptosis in human cells from tumors through the inhibition of Ras signaling pathways [74, 75]. Statins also promote autophagy through the activation of the AMPK-TOR signaling pathway in cells from rhabdomyosarcoma [76]. Treatment with lovastatin increases the expression of Rab7 mRNA by decreasing the synthesis of isoprenyl groups and promoting phagosome maturation [77].

### 2.1. Effect of Statins on Infectious Diseases

With respect to statins and their antimicrobial effect, it has been reported that statin therapy reduces mortality in patients with bacteremia and multiple organ failure [78]. Several studies have evaluated the benefit of the use of statins in the prevention or treatment of sepsis, although some results are contradictory. In different meta-analyses, promising results have been observed in which treatment with statins significantly reduced the progression of the disease and/or mortality associated with sepsis [79–81].

*In vitro* studies have shown some antimicrobial effects against gram-positive and gram-negative bacteria and on some viruses and fungi [82]. The addition of atorvastatin or lovastatin reduces the *in vitro* growth of *Chlamydophila pneumoniae* [83] and of *Salmonella enterica* [84]. Simvastatin demonstrated a significant antimicrobial effect against methicillin*-*sensitive *Staphylococcus aureus* (average MIC, 15.65 *μ*g/mL) and, to a lesser extent, against methicillin*-*resistant *S. aureus* (MIC 31.25 *μ*g/mL), inhibiting adhesion and formation of biofilm of the microorganism [85].

It has been observed that lovastatin interferes with the replication of hepatitis C virus RNA through the inhibition of geranylgeranylation protein of the host [86]. Statins also inhibit the assembly of dengue virus virions through a mechanism independent of cholesterol levels [87]. Statins have also shown antiviral effects on cytomegalovirus [88], the Epstein-Barr virus [89] and HIV infection [90]. The antiviral mechanism that statins exert is not clear; however, the metabolite rescue experiments suggest participation of the nonsteroidal isoprenoid arm of the mevalonate pathway as a possible mechanism of action [91, 92].

Statins inhibit the formation of biofilms of *Candida albicans* [93] and, in *C. glabrata*, reduce ergosterol levels, inhibit their growth, and cause the loss of mtDNA [94]. Statins also inhibit the growth of *Aspergillus fumigatus*; in addition, lovastatin strengthens the activity of caspofungin against *A. fumigatus* in an *in vitro* model [95, 96].

Together, these *in vitro* studies show that statins slow the growth of some microorganisms, including some resistant bacteria, and also show that they can interfere with biofilm formation. These effects have clinical relevance because within the biofilm, bacteria are protected against the action of antibodies, the attack of phagocytic cells, and the effect of antimicrobials. It is also known that biofilm bacteria can be up to 1000 times more resistant to antibiotics than the same bacteria grown in liquid medium [97].

Therefore, it is very important to note that statins have the potential to inhibit the growth of resistant bacteria and interfere with the biofilm formation process. However, it is necessary to emphasize that the concentrations used in *in vitro* studies that have antimicrobial properties are 100 to 1000 times higher than the plasma concentrations reached in patients undergoing statin therapy, which is why it is still necessary to clarify whether these effects are transferable and if they have any therapeutic benefit in humans.

### 2.2. *In Vitro* Effect of Statins on *Mycobacterium tuberculosis* Infection

The first study on the potential effect of statins on infection by *M. tuberculosis* was performed 18 years ago by Montero et al., where it was observed that fluvastatin slightly induces the release of TH1 cytokines and promotes the activation of caspase 1; by infecting peripheral blood mononuclear cells (PBMCs) with *M. tuberculosis* and treating them with fluvastatin, this stimulation was synergistic, yielding concentrations up to 10 times higher than those of TH1 cytokines and caspase 1. This result suggested that statins could strengthen the host response against *M. tuberculosis* [67]. In 2009, another study reported that lovastatin and fluvastatin inhibit the activation of *γδ* T cells induced by *M. tuberculosis* antigens [98]; however, none of these studies evaluated the effect of statins on mycobacterial growth or the influence of these effects on the immune response of the host against infection (Table 1).

In 2014, Parihar et al. [99] found that mononuclear cells and monocyte-derived macrophages from patients with familial hypercholesterolemia who had received statin therapy for at least six months were more resistant to infection with *M. tuberculosis* with a multiplicity of infection (MOI) of 5 (number of bacteria per number of human/mammal cells) compared with cells from healthy subjects who had never taken statins. In the same study, they performed an *in vitro* model with murine bone marrow-derived macrophages that were exposed to simvastatin at a concentration of 50 *μ*M and were infected with *M. tuberculosis* at an MOI of 5. The results showed a significant reduction in mycobacterial growth, without adverse effects on cell viability. In addition, they performed Western blot and confocal microscopy experiments, where it was observed that simvastatin promotes maturation in phagosomes and autophagy in macrophages infected by *M. tuberculosis*. The effect of simvastatin on the inhibition of *M. tuberculosis* growth was reversed by mevalonate supplementation (Table 1). This was the first study where the use of statins as HDT was proposed, and the study opens the possibility of studying the additive effect of statins with first-line drugs in drug therapy against tuberculosis.

Lobato et al. investigated the activity and the possible additive effects of treatment with rifampicin (1 *μ*g/mL), atorvastatin (0.2 *μ*M-2 *μ*M), and simvastatin (0.2 *μ*M-2 *μ*M) on THP-1 macrophages infected with *M. tuberculosis*, BCG strain of *M. bovis*, and *M. leprae* at an MOI 10. After 72 hours, both statins had dose-dependent bactericidal effects on all strains. For *M. tuberculosis*, both statins (2 *μ*M atorvastatin and 2 *μ*M simvastatin) reduced the viability of the mycobacteria by approximately 75% and showed an additive effect with rifampicin (1 *μ*g/mL rifampicin plus 0.2 *μ*M atorvastatin or 0.2 *μ*M simvastatin). For the BCG strain of *M. bovis* and *M. leprae*, only 0.2 *μ*M atorvastatin had an additive effect with rifampicin. To determine the mechanism involved in the inhibition of mycobacterial growth, they only tested the effect of atorvastatin on THP-1 macrophages infected with *M. leprae* at an MOI of 10; the results confirmed that statins promote phagosome maturation (Table 1) [100].

Another study investigated whether statins could strengthen the bactericidal effect of isoniazid. They tested J774 macrophages, which were infected with *M. tuberculosis* CDC1551 at an MOI of 10, and treated them with 5 *μ*M simvastatin and 0.05 *μ*g/mL isoniazid. The results confirmed that cells cultured in the presence of simvastatin and infected with *M. tuberculosis* CDC1551 had a lower intracellular bacillary load on day five after infection, and this effect was additive when the cells were treated simultaneously with simvastatin and isoniazid on the third day after infection. On the fifth day, the additive effect lost statistical significance [101].

In 2016, Dutta et al. studied the possible adjuvant activity of simvastatin with isoniazid, rifampicin, and pyrazinamide. They performed experiments with the *M. tuberculosis* H37Rv expressing the *lux* operon and THP-1 macrophages infected at an MOI of 0.05. They used drugs at concentrations used in experiments to reduce 50% of the relative light units (RLU) of the mycobacterium (0.011 *μ*M isoniazid, 0.012 *μ*M rifampicin, and 162.5 *μ*M pyrazinamide); the simvastatin concentration was 0.1 *μ*M. The results confirmed that simvastatin without antibiotic inhibited the growth of mycobacteria in the THP-1 macrophages compared with the control without drugs, and the effect of simvastatin was equivalent to the activity of 0.011 *μ*M of isoniazid. In addition, simvastatin significantly increased the bactericidal effect when the three drugs were added simultaneously [7]. They also evaluated whether simvastatin could affect the intracellular accumulation of rifampicin using liquid chromatography-mass spectrometry. It was observed that although simvastatin (0.1-1 *μ*M) increased the bactericidal activity of rifampicin, it did not alter its intracellular accumulation (Table 1) [7].

The different studies used different strains of mycobacteria (reference and clinical isolates) and different MOIs (from 0.05 to 10); however, the effect of statins has been consistent, and the studies have shown that statins activate several cellular mechanisms (autophagy and maturation of the lysosome phage) by which control of infection in infected cells is favored *in vitro*. However, identification of the possible direct antimicrobial effect of statins has not been performed.

Skerry et al. reported the first study on the direct antimicrobial effect of simvastatin against *M. tuberculosis* tested at different concentrations (0-320 *μ*M). The results obtained showed that simvastatin had no inhibitory effect, even at the highest concentration (320 *μ*M, a concentration 60 times higher than those used in *in vitro* experiments performed in cells) [101].

Another study also evaluated the direct antimicrobial effect of statins on *M. tuberculosis* H37Rv and the BCG strain of *M. bovis* using the agar proportion method, with different concentrations of simvastatin. The MIC found was 100 *μ*g/mL (238.91 *μ*M), defined as the lowest concentration of the statin that inhibited more than 99% of the bacterial population. In addition, this study reported that simvastatin decreases the amount of phosphatidylinositol mannosides and triacylglycerols present in the mycobacterial cell wall (Table 1) [102].

The MICs reported by the CLSI for the first-line drugs are as follows: isoniazid, 0.2 or 1.0 *μ*g/mL; rifampin, 1.0 *μ*g/mL; pyrazinamide, 100 *μ*g/mL; and ethambutol, 5.0 *μ*g/mL [103]. In contrast, the MIC for simvastatin is elevated and similar to that of pyrazinamide, well above the therapeutic concentrations recommended for the treatment of hyperlipidemia (0.0209 *μ*g/mL) [104]. Thus, the direct antimicrobial effect *in vitro* appears to be weak, which supports the idea that simvastatin favors or triggers the cellular mechanisms necessary to eliminate the bacillus.

### 2.3. Treatment of Tuberculosis with Statins in Animal Models

All *in vivo* and prospective studies of the effect of statins against tuberculosis have been performed in mice (Table 2). The first study was performed in C57BL/6 mice, 8-12 weeks of age, treated intraperitoneally with simvastatin or rosuvastatin (20 mg/kg) or with phosphate-buffered saline (PBS) as a control every two days for two weeks; the mice were infected with *M. tuberculosis* by exposure to aerosols. Statin therapy was continued until four weeks after infection. Both statins showed a protective response in the infected mice, with up to 10-fold reductions in the bacillary load in the spleen, liver, and lungs of mice infected and treated with simvastatin, in comparison with untreated control animals (Table 2) [99]. Lobato et al. investigated the effect of atorvastatin using the Shepard infection model [105], in which a suspension of 1 × 10^4^*M. leprae* was injected into the plantar pads of BALB/c mice. After one month of infection, the mice were treated with atorvastatin (80 mg/kg/day) in combination with rifampicin (1 mg/kg) for five months. The results showed that atorvastatin reduced the replication of *M. leprae* and had an additive effect with rifampicin. They also showed that treatment with atorvastatin or the combination of rifampin plus atorvastatin did not increase muscle damage or hepatotoxicity in mice (Table 2) [100].

Subsequently, another group tested whether the addition of statins to the standard first-line treatment regimen could increase bactericidal capacity. BALB/c mice four to six weeks of age were infected by aerosols with 3.7 log10 CFU of *M. tuberculosis* CDC1551. The infection was allowed to progress for six weeks before the start of treatment, after which the mice were treated by gavage with rifampicin (10 mg/kg), isoniazid (10 mg/kg), and pyrazinamide (25 mg/kg), with or without simvastatin (25 mg/kg), five days per week for eight weeks. After four and eight weeks of treatment, the standard regimen in combination with simvastatin showed a greater efficacy for the elimination of mycobacteria, reducing the number of lung CFU by one additional log10 on day 28 and 1.25 log10 on day 56 [101], suggesting that simvastatin could improve current treatment (Table 2).

Dutta et al. confirmed that simvastatin therapy as an adjuvant to standard treatment reduced the time to obtain a negative culture in BALB/c mice infected with *M. tuberculosis* H37Rv by aerosols. They also evaluated the relapse rates in mice treated with simvastatin (60 mg/kg) for 2.5, 3.5, and 4.5 months. Relapse was evaluated three months after stopping treatment. The results showed that treatment with anti-TB drugs plus simvastatin reduced the percentage of relapses by 50% compared with treatment with only anti-TB drugs (Table 2) [7].

These studies together propose statins as adjuvant treatment to first-line drugs for the treatment of active tuberculosis, as they show that treatment with statins reduces the bacillary load, shortens the duration of therapy, and decreases the relapse rate. However, these studies have some limitations. First, the doses used in mice are much higher than those used in humans. Second, some statins are prodrugs, and in mice, 100% of the prodrug is converted into the active metabolite, whereas the conversion reaches 50% of the administered dose in humans [106]. It is also necessary to determine the best time to start treatment with statins, as the treatment started before infecting the mice in the studies reported. In addition, it would be advisable to carry out studies in other animal models that have a drug metabolism more similar to that of humans and to determine the concentrations associated with better antimicrobial activity.

### 2.4. Retrospective Studies of Statin Use in Humans and the Risk of Developing Tuberculosis

Kang et al. reported in 2014 the first study in humans that evaluated the effect of statins on the risk of developing tuberculosis. This study was retrospective, and the results were obtained from the information of the database of the National Health Insurance of South Korea, which included 840,894 subjects with recently diagnosed diabetes mellitus type 2 (DM2) and who were 20-99 years of age. The use of statins was less frequent among patients with active tuberculosis (19.2% patients with tuberculosis vs. 33.6% without tuberculosis, *p* < 0.01). However, after adjustment for possible initial confounding factors (age, sex, history of silicosis, malignancy, HIV/AIDS, chronic kidney disease, use of systemic corticosteroids, comorbidities [e.g., dyslipidemia, hypertension, angina, myocardial infarction, cerebrovascular disease, peripheral artery disease, and retinopathy], and history of hospitalization), the use of statins in subjects with newly diagnosed DM2 was not associated with a lower or higher risk of developing tuberculosis (hazard ratio [HR] 0.98, 95% CI 0.89-1.07) [107]. The authors note that although comorbidities were adjusted as confounding variables, they could influence the HR for active tuberculosis development (Table 3).

Subsequently, another retrospective study conducted by Lee et al. in Taiwan included 13,981 patients with DM2 older than 65 years. They used the Cox proportional hazards regression model to determine the independent effects of diabetes on the risk of developing active tuberculosis. After adjusting for age, sex, comorbidities (gout, hypertension, hyperlipidemia, asthma, COPD, acquired immunodeficiency syndrome (AIDS), connective tissue disease, end-stage renal disease, heart failure, and other cardiovascular diseases) and medications (antihyperglycemic, antihypertensive, and antihyperlipidemic agents), the investigators reported that Taiwanese diabetic subjects older than 65 treated with statins had a lower risk of developing active tuberculosis, with a risk of 0.76 (95% CI, 0.60-0.97) [108]. In this study, exposure to statins was based only on the prescription information compiled from the National Health Insurance Database; therefore, the level of adherence and dose received by patients is unknown (Table 3).

Lai et al. conducted a nested case-control study that included patients older than 18 years of age from 1999 to 2011, using the database from Taiwan's national health insurance program. They included 8098 new cases of tuberculosis and 809,800 control patients, and statin users were divided into four groups. The first group was called the current users (patients with prescription of statins within 30 days before the diagnosis of tuberculosis), the second group were the recent users (patients with prescription of statins between 31 and 90 days before the diagnosis of tuberculosis), the third group were patients with statin consumption between 91 days and one year before the date of tuberculosis diagnosis, and the fourth group were those with chronic use (patients with a cumulative prescription greater than 90 days). They used a sampling strategy to control cases of coincident time, and the relative risk (RR) of developing active tuberculosis was calculated with a confidence interval greater than 95%. The four types of statin users had a lower risk of active tuberculosis. For the first group, the RR of developing active tuberculosis was 0.64 (95% CI 0.54-0.76). The fourth group showed the lowest risk of developing active tuberculosis (RR: 0.62, 95% CI: 0.53 to 0.72) [109]. One of the methodological strengths of this study is the inclusion of a large sample of patients and controls and the performance of a conditional logistic regression analysis with more than 75 possible confounding factors (e.g., cardiovascular comorbidities, risk factors for developing tuberculosis, frailty indicators, and use of specific medications). However, some data related to lifestyle and its effects (e.g., diet, exercise, overcrowding, smoking, and body mass index) are not available; therefore, the effects of some residual confounding variables are unknown (Table 3).

Another case-control study also conducted in Taiwan included 8236 subjects older than 20 years of age with recently diagnosed pulmonary tuberculosis from 2000 to 2013. Each case was matched by age and sex with 8236 controls without pulmonary tuberculosis. The users of some type of statin were subjects who received medication 12 months before being diagnosed with pulmonary tuberculosis. Multivariable logistic regression analysis was performed to estimate the odds ratio (OR) with a confidence interval of 95%. After adjusting for lipid-lowering drugs that were not statins and for DM2, the OR adjusted for pulmonary tuberculosis was 0.67 for the subjects who used statins at some point (95% CI: 0.59 to 0.75). They also analyzed the estimated OR for each type of statin, and the results showed that the subjects taking atorvastatin had a lower probability of developing active tuberculosis (0.56, 95% CI 0.46, 0.68) [110]. The researchers confirmed that the use of statins is a protective factor for the development of pulmonary tuberculosis. However, like all studies conducted using a database, there are residual confounding factors due to a lack of information. In this study, we did not have access to socioeconomic status and lifestyle data (Table 3).

The most recent published study (also retrospective) included 102,424 statin users and 202,718 control subjects (no statin use). The use of statins was defined as a prescription record for ≥30 days of some type of statin. They calculated the cumulative defined daily dose (cDDD) of statins and defined three groups: low (<180), medium (180-365), and high (>365). The HR for the development of tuberculosis disease in patients taking statins was 0.53 (95% CI: 0.47 to 0.61, *p* < 0.001), suggesting that the use of statins is an independent protection factor for the development of tuberculosis. They also found a dose-dependent association between the use of statins and the risk of active tuberculosis (low, HR 1.06, *p* = 0.477; medium, HR 0.57, *p* < 0.001; high, HR 0.27, *p* < 0.001) [111]. In this study, the authors adjusted the possible confounding variables, including age, sex, level of urbanization, visits to the emergency department, comorbidities (such as DM2, coronary heart disease, heart failure, cerebrovascular disease, chronic kidney disease, cancer, lung disease, asthma, liver cirrhosis, rheumatoid arthritis and systemic lupus erythematosus), and the use of medications (biological agents, systemic glucocorticoids, and disease-modifying antirheumatic drugs). However, again, information regarding the diagnosis of tuberculosis and the use of statins depended on a database; thus, the study suffers from the lack of precision that can be achieved with a prospective cohort study (Table 3).

All the studies carried out on the effect of statins on the risk of developing tuberculosis have been performed in Korea and Taiwan, so the generalization of the results to other populations requires verification. In addition, since they are retrospective studies obtained from databases, no microbiological data are available to support the diagnosis of tuberculosis, and a causal relationship cannot be verified.

## 3. Perspectives and Conclusions

The information gathered in this review provides the basis for considering statins as a host-directed therapy in infection by *M. tuberculosis*. The studies described show that the cells (mostly macrophages) are more resistant to infection by *M. tuberculosis* in the presence of statins in *in vitro* models. The mechanism proposed to favor the immune response of the host is promoting phagolysosome maturation and autophagy. However, it is necessary to deepen the knowledge of the effect of statins on the immune response against *M. tuberculosis*. In this sense, our group recently reported that treatment with simvastatin increases the percentage of NKT cells and increases the expression of costimulatory molecules in monocytes in an infection model *in vitro*, and the increase in the expression of these molecules may favor the antigenic presentation that is inhibited by *M. tuberculosis* [112].

Studies in mice show that statin therapy shortens the duration of antituberculosis treatment and appears to decrease the risk of relapse. Therefore, investigations of the mechanism through which statins increase the antimicrobial effect of first-line antituberculosis drugs are still needed, although we can speculate that it is possible that statins weaken the mycobacterial wall making it more susceptible to first-line drugs, that they may only strengthen the immune response of the host that contributes to the most effective and rapid elimination of the bacillus, or even a combination of both effects; specifically, it has been shown that statins affect the lipids of the wall of some fungi and can also decrease the phosphatidylinositol mannosides and triacylglycerols of the cell wall of *M. tuberculosis* H37Rv.

Although the mechanism by which statins improve the immune response against *M. tuberculosis* is still not fully known, retrospective studies in humans show a protective effect of statins against the development of active tuberculosis. The evidence that exists to date is sufficient to test statins in prospective studies for the determination of whether these drugs have only protective effects against the reactivation of latent tuberculosis or if they could really be effective adjuvants in the pharmacological treatment of active tuberculosis.

## Figures and Tables

**Table 1:** *In vitro* effects of statins in tuberculosis.

Author	Cell type	Treatment	Strain	Effect
Montero et al.	PBMC	Fluvastatin 5 *μ*m	Heat-inactivated *M. tuberculosis* H37Ra 10 *μ*g/mL	Promotes release of TH1 cytokines and promotes the activation of caspase 1
Lu et al.	PBMC	Lovastatin 10 *μ*m Fluvastatin 2 *μ*m	*M. tuberculosis* antigens	Inhibits the activation of *γδ* T cells
Parihar et al.	PBMC and MDM from patients with hypercholesterolemia receiving statin therapy	Simvastatin 50 *μ*M	*M. tuberculosis* H37Rv MOI 5	Significant reduction of mycobacterial growth
Parihar et al.	Murine bone marrow-derived macrophages	Simvastatin 50 *μ*M	*M. tuberculosis* H37Rv MOI 5	Significant reduction of mycobacterial growth, simvastatin treatment promotes phagosomal maturation and autophagy
Lobato et al.	THP-1 macrophages	Rifampin 1 *μ*g/mL plus 0.2 *μ*M atorvastatin	*M. tuberculosis* H37Rv MOI 10 BCG strain of *M. bovis* MOI 50	Atorvastatin has an additive effect with rifampin, reducing intracellular mycobacterial viability
Skerry et al.	J774 macrophage-like cells	Isoniazid 0.05 *μ*g/mL plus 5 *μ*M simvastatin	*M. tuberculosis* CDC1551 MOI 10	Simvastatin treatment enhanced the bacterial killing activity of isoniazid at day 3 after infection
Dutta et al.	THP-1 macrophages	0.011 *μ*M isoniazid, 0.012 *μ*M rifampicin, and 162.5 *μ*M pyrazinamide plus 0.1 *μ*M simvastatin	Bioluminescent *M. tuberculosis* H37Rv MOI 0.05	Simvastatin treatment significantly increased the bactericidal effect of isoniazid, rifampicin, and pyrazinamide alone or in combination

PBMC: peripheral blood mononuclear cells; MDM: monocyte-derived macrophages.

**Table 2:** Statin treatment of tuberculosis in animal models.

Author	Animal model	Treatment	Strain	Effect
Parihar et al.	C57BL/6 mice (age 8-12 weeks)	Simvastatin or rosuvastatin (20 mg/kg) i.p. every second day for 6 weeks	Low-dose aerosol-based *M. tuberculosis* H37Rv	Up to a 10-fold reduction in bacilli burden in spleen, liver, and lungs
Lobato et al.	Mouse foot pads of BALB/c mice (Shepard's mouse model)	Atorvastatin (80 mg/kg/day added daily to food) alone or combined with rifampin (1 mg/kg by gavage weekly) for five months	1 × 10^4^ live *M. leprae* in 10 *μ*L inoculated in each footpad	Reduced replication, additive effect with rifampin. Neither atorvastatin treatment nor combination treatment increased muscle damage or induced hepatotoxicity
Dutta et al.	BALB/c mice (age 4-6 weeks)	Rifampicin (10 mg/kg), isoniazid (10 mg/kg), and pyrazinamide (25 mg/kg), plus simvastatin (25 mg/kg) by gavage 5 days per week for 8 weeks	Aerosol infection with 3.7 log10 CFU of *M. tuberculosis* CDC1551	The combination regimen with simvastatin enhanced mycobacterial killing and reduced the relapse rates in mice treated for 2.5 and 3.5 months

**Table 3:** Retrospective studies of statin use in humans and the risk of developing tuberculosis.

Author	Year	Method	Patients	Conclusions
Kang et al.	2014	Retrospective cohort study	840,894 newly diagnosed type 2 DM patients aged 20-99 years who were newly treated with antidiabetic drugs	Statin use in newly diagnosed type 2 diabetics was not associated with protection against or a higher risk of developing tuberculosis
Lee et al.	2015	Retrospective cohort study	13,981 patients with type 2 diabetes aged more than 65 years	After adjusting for age, sex, other comorbidities, and medications, statin users had a lower independent association, with a risk ratio of 0.76 (95% CI, 0.60-0.97)
Lai et al.	2016	Retrospective nested case-control study	8098 new TB cases and 809,800 control patients	Statin users had a decreased risk of active tuberculosis. Chronic use of statins (more than 90 days) was associated with the lowest risk (RR 0.62; 95% CI 0.53-0.72)
Su et al.	2017	Retrospective nested case-control study	102,424 statin users, 202,718 patients aged 20 years or older, and 202,718 matched subjects	Statin use is an independent protective factor for tuberculosis development. There is a dose-dependent association between statin use and risk of active tuberculosis
